# The positive impact of a care–physical activity initiative for people with low socioeconomic status

**DOI:** 10.1093/eurpub/ckac130.007

**Published:** 2022-10-25

**Authors:** LS Mulderij, KT Verkooijen, AS Groenewoud, MA Koelen, MAE Wagemakers

**Affiliations:** 1 Health and Society, Wageningen University & Research, Wageningen, Netherlands; 2 IQhealthcare, Radboudumc, Nijmegen, Netherlands

## Abstract

**Background:**

Overweight and obesity rates are increasing worldwide, particularly among people with a low socioeconomic status (SES). Care-physical activity (care-PA) initiatives may lower overweight and obesity rates. A two-year care-PA initiative specifically developed for citizens with a low SES, X-Fittt 2.0, included 12 weeks of intensive guidance and sports sessions, and 21 months of aftercare. We answered the research question: what are the short- and long-term outcomes of participation in X-Fittt 2.0 in terms of health, quality of life and societal participation?

**Methods:**

Questionnaires and body measurements were taken from 208 participants at the start of X-Fittt 2.0 (t0) and after 12 weeks (t1), 1 year (t2) and 2 years (t3). We also held 17 group discussions (t1, n = 71) and 68 semi-structured interviews (t2 and t3). Continuous variables were analysed using linear mixed-model analysis, while we used descriptive statistics for the categorical variables. Qualitative data were analysed using thematic analysis.

**Results:**

Body weight was significantly lower at all three post-initiative time points compared with t0, with a maximum of 3.8 kg difference at t2 (p < 0.05). BMI, waist circumference, blood pressure and self-perceived health only significantly improved during the first 12 weeks (p < 0.05). A positive trend regarding paid work was observed, participants reported increased PA levels (including sports) and a few stopped smoking or drinking alcohol. Participants felt healthier and more energetic, reported improved self-esteem and stress levels, and had become more socially active. However, barriers to being physically active included a lack of money or time, or physical or mental health problems.

**Conclusions:**

X-Fittt 2.0 improved the physical health, QoL and societal participation of the participants. Future initiatives should take into account the aforementioned barriers, and consider a longer intervention period for more sustainable results.

**Key messages:**

- Care-physical activity initiatives can improve the physical and mental health, quality of life, lifestyle and societal participation of citizens with a low socioeconomic status.
- It is vital to improve the accessibility of care-physical activity initiatives, for example by lowering costs, so that existing and future initiatives better suit people with low socioeconomic status.

